# LC–MS/MS-Based Quantification Method of Polyphenols for Valorization of Ancient Apple Cultivars from Cilento

**DOI:** 10.1021/acsfoodscitech.1c00439

**Published:** 2022-03-30

**Authors:** Anna Illiano, Gabriella Pinto, Maria Antonietta Carrera, Angelo Palmese, Riccardo Di Novella, Paolo Casoria, Angela Amoresano

**Affiliations:** † Department of Chemical Sciences, University of Naples Federico II, 80126 Naples, Italy; ‡ CEINGE Advanced Biotechnologies, University of Naples Federico II, 80145 Naples, Italy; § INBB, Istituto Nazionale Biostrutture e Biosistemi, Consorzio Interuniversitario, 00136 Rome, Italy; ∥ Ecomuseo della Valle delle Orchidee e delle Antiche Coltivazioni-Sassano (Sa)-PNCVDA, 84038 Sassano, Italy; ⊥ Department of Sciences and Technology, University of Naples Parthenope, 80143 Naples, Italy; # Pharmaceutical & Analytical Development Biotech Products, Merck Serono SpA, an affiliate of Merck KgaA, Darmstadt, Germany, 00176 Roma, Italy

**Keywords:** polyphenols, Campania region, safeguarding of ancient agricultural traditions, mass spectrometry

## Abstract

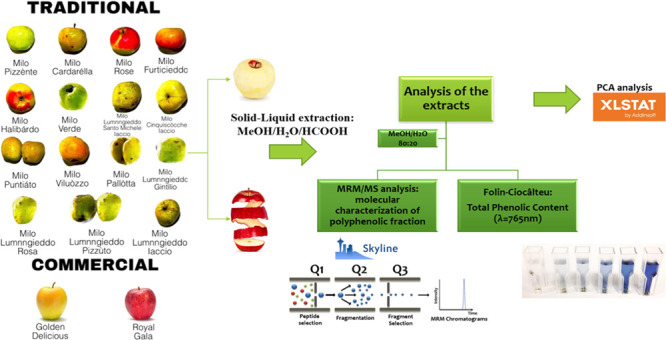

Safeguarding the biodiversity of plant species is of fundamental importance for their defense against pests and diseases even through the maintenance and dissemination of ancient agricultural traditions rooted within the small rural environments. The investigation area of the current research covered some municipalities belonging to the “Parco Nazionale del Cilento e Vallo di Diano” including the sub-mountainous part of “Comunità Montana del Vallo di Diano (Salerno, Campania)”. Fifteen ancient apple varieties were collected from local communities to be analyzed and compared to some commercially available apples. To this aim, a Folin–Ciocâlteu assay was preliminarily used to measure the total polyphenol content in both ancient and commercial apple cultivars. Then, a liquid chromatography−tandem mass spectrometry (LC–MS/MS) analysis in the multiple reaction monitoring (MRM) ion mode was then implemented to detect and quantify specific polyphenols and to obtain a molecular comparison of a wide panel of polyphenols. The main finding of the present work pointed out that ancient apple cultivars are richer than commercial ones in anthocyanins, dihydrochalcones, and chlorogenic acid, whose beneficial effects on health are widely known. Thus, the safeguarding of these ancient varieties is greatly encouraged for the richness of polyphenols crucial both for the defense of plants from insects and for remarkable nutraceutical properties, in addition to the need for germplasm conservation as a source of genetic variability.

## 1 Introduction

The presence of ancient varieties of fruit trees represents a great source of genetic variability supporting the artificial selection during the centuries of cultivation. Actually, safeguarding the rural environments through the maintenance and dissemination of ancient agricultural traditions, is of fundamental importance to avoid the depletion of biodiversity, which is a cornerstone of the environmental policy in the geographical area of interest. The gradual abandonment of the ancient crops is due to the expansion of the industrial fruit cultivation and to the predilection for fruits with regular shapes and sizes in agreement to the standardization criteria of the national market. In the Campania region, traditional cultivations of a high number of cultivars, for example, apples, pears, plums, and so forth, are locally preserved in small areas. The recovery of these ancient fruits is of scientific interest as it allows to conserve the germplasm and thus to protect the entire autochthonous diversity.

The investigation area of the current research covers the territories of some municipalities belonging to the “Parco Nazionale del Cilento e Vallo di Diano” and to the “Comunità Montana del Vallo di Diano (Salerno, Campania)”, a sub-mountainous area at the foot of Mount Cervati, between 650 and 750 m a.s.l. This geographical territory has already been of precious interest to some studies on medicinal and food species^1^ in addition to the identification of ancient fruit species. Moreover, this area owes its popularity because of the first studies on the so-called “good Mediterranean diet” carried out by Ancel Keys.^2^ From a demographic point of view, this is a rather isolated area, with mainly well-preserved agro-ecosystems, a virtually intact agricultural and rural landscape, and a body of local traditions and material culture well-characterized. The fragmentation of the cultivations allowed maintaining agricultural practices still related to the ancient agricultural techniques and to the ecotypes that in these places had demonstrated better adaptability.

The varieties of apples under investigation are peculiar to Cilento with peasant communities partaking of the diet of rural populations. The origin of these varieties is not well-known, and the diffusion on the territory seems to be associated with the transhumance, during which the shepherds grafted the apples they cultivated on wild plants they found along the way, with the intention of being able to supply them in the years to come during their journey.

Since the 1990s, particular attention has been focused on the content of bioactive compounds in foods, especially on the phenolic component of fruits, with the aim to reveal their potential contribution to health and the preservation of food quality.^3^ Moreover, great effort has been made for the development of functional food derivatives that can confer positive health—beyond basic nutrition to consumers. The wider scientific literature demonstrates that a diet rich in polyphenols, such as flavonoids and phenolic acids, have a crucial role in antioxidative, antiaging, antibacterial, and antimutagenic properties.^4^

The study focused on polyphenol characterization of fifteen varieties of apples five of which probably derived from a variety still widely cultivated in Campania, *Limoncella* apples (e.g., Milo Limungieddo *Iaccio, Milo Limungieddo Gintilo, Milo Limungieddo Pizzuto, Limungieddo Santomichele Iaccio,* and *Milo Limungieddo Rosa*) and two varieties (e.g., *Milo Cinquiscocche Iaccio* and *Milo Limungieddo Iaccio*) possibly derived from crosses between the *Limoncella* apple and *Malus astracanica*, Analyzing these ancient varieties has the dual goal of measuring their total polyphenol content (TPC) in order to compare them with the commercial ones and of encouraging a possible return to the market as native products of value. Moreover, the detection and quantification of specific polyphenols by using the liquid chromatography tandem mass spectrometry (LC–MS/MS) method in the multiple reaction monitoring (MRM) ion mode have further pointed out that ancient apple cultivars are a valuable source of polyphenols crucial both for the defense of plants from insects and for remarkable nutraceutical properties, in addition to the need for germplasm conservation as a source of genetic variability.^5^

## 2 Materials and Methods

### 2.1 Chemicals

Polyphenol standards (malvidin-3-*O*-glucoside, naringin, catechin, quercetin, gallic acid, vanillic acid, caffeic acid, and ferulic acid), methanol, acetic acid, the Folin–Ciocâlteu (FC) reagent, gallic acid, and sodium carbonate were purchased from Merck (Darmstadt, Germany); 2-propanol and acetonitrile (ACN) were purchased from Honeywell (Charlotte, USA); and formic acid was supplied by J.T. Baker (Rodano, Italy).

### 2.2 Collection of Samples: Traditional and Commercially Available Apple Cultivars

**Figure 1:**
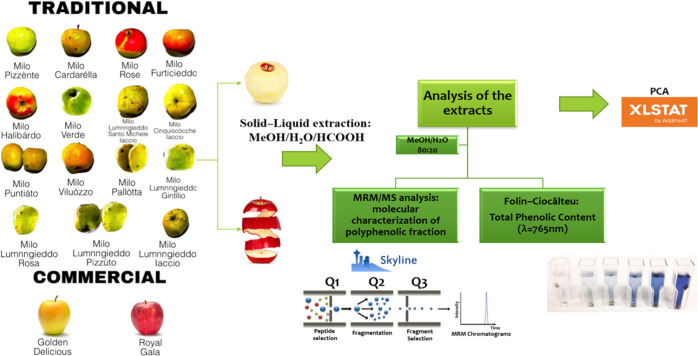
Experimental strategy for polyphenol characterization of ancient and commercially available apple cultivars.

Fifteen traditional apple cultivars: “*Milo Cardarella*”, “*Milo Cinquiscocche Iaccio*”, “*Milo Halibardo*”, “*Milo Limungieddo Iaccio*”, “*Milo Limungieddo Gintilo*”, “*Milo Limungieddo Pizzuto*”, “*Milo Limungieddo Santomichele Iaccio*”, “*Milo Limungieddo Rosa*”, “*Milo Pallotta*”, “*Milo Pizzente*”, “*Milo Puntiato*”, “*Milo Furticieddo*”, “*Milo Rose*”, “*Milo Verde*”, and “*Milo Viluozzo*” were collected at the maturity stage in 2019, November, from “*Ecomuseo della valle delle orchidee e delle antiche coltivazioni di Sassano*” in Italy. The commercial apple cultivars “*Golden Delicious*” and “*Royal Gala*” were purchased from the local fresh market (Figure 1). The provided samples were frozen at −20 °C to obtain better storage conditions, and they were defrosted 16 h before the polyphenols’ extraction was performed.

### 2.3 Solid–Liquid Extraction

The extraction of polyphenols and other metabolites was carried out by collecting samples of the peel and pulp from the apples by using a scalpel. These samples were weighed and added to the extraction solvent (methanol/water/formic acid 80:19:1) in a weight (g)/volume (mL) ratio of 1:3.

The samples were homogenized using an ULTRA-TURRAX T25 (IKA, Staufen, Germany), sonicated (ELMAsonic s30, Elma Ultrasonic Technology, Singen, Germany) for 15 min at room temperature while the sample was kept refrigerated in ice, and centrifuged for 10 min at 5000 rpm, and the liquid fraction was filtered on Macherey-Nagel CHROMAFIL Xtra PTFE-45/25 syringe filters. The filtered extracts were dried under nitrogen and solubilized in 2 mL of extraction mixture for subsequent analysis.

### 2.4 Total Phenolic Content: FC Assay

The FC assay was performed on the apple raw extracts according to the method of Benelli et al. (2010).^6^ In each cuvette, 790 μL of MilliQ water, 150 μL of a 20% Na_2_CO_3_ solution, 50 μL of the FC reagent, and 10 μL of apple raw extract (both peel and pulp) solubilized in a solution of methanol/water/formic acid with a ratio of 80:19:1 were added. The cuvettes were kept in the dark for 2 h, and then, the spectrophotometric analysis was performed by recording for each sample the absorbance at 765 nm. Gallic acid (0.25–2 g/L) was used as a standard for the calibration and construction of a linear regression line. A solution of methanol/water/formic acid with a ratio of 80:19:1 was used as the blank. The total phenolic content (TPC) is expressed as milligrams of gallic acid equivalent (GAE) in 100 g of the sample.

### 2.5 Targeted LC–MS/MS Analysis

LC was performed on an LC Eksigent operating with a column Halo C18 2.7 μm 90A 1 × 50 mm (Munich, Germany) at a temperature of 45 °C. The elution was performed during a total run of 8.5 min at a flow rate of 40 μL/min using a mobile phase A containing 0.1% formic acid and 5 mM ammonium formate in water and a mobile phase B consisting of 0.1% formic acid and 5 mM ammonium formate in ACN. The gradient table for the LC run provided the following gradient: 0–1 min at 1% B; 1–3 min at 12% B; 3–6 min at 20% B; 6–8 min at 99% B; and 8–8.5 min at 1% B.

The MS analysis was performed on a 4000 QTRAP from AB Sciex (Darmstadt, Germany), equipped with an electrospray ionization source and a hybrid triple quadrupole LIT (linear ion trap) designed for qualitative and quantitative purposes. The analyses were carried out in the positive negative^7^ and positive MRM ion mode as reported in Supplementary Table 1 (Table S1). Electrospray source parameters such as curtain gas (CUR), collision gas (CAD), ion spray voltage (IS), and source temperature (TEM) were set to be 20.0 psi, 5 psi, 4.5 kV, and 380.0 °C, respectively. According to Pinto^7^ et al. and Lo Piccolo^9^ et al., quantitative analyses were performed by using the external standard method, and calibration curves were realized in the 1–500 pg/μL range of concentrations.^7−9^

The interpretation of the targeted data was realized using Skyline software 20.2.0.343 version (MacCoss Lab, Department of Genome Sciences, UW). By importing the .wiff files, the software was used to process the multireplicate data obtained for the standards and the samples. The chromatographic peaks corresponding to each compound were identified, and the peak areas were extracted and interpolated on calibration curves, accordingly to structural homology, to obtain the compound concentration expressed as μg/g of fresh weight (FW).

### 2.6 Principal Component Analysis

The values of target analyte concentrations were assessed as quantitative variables and apple cultivar names were considered as observations to perform statistical analysis. XLSTAT statistical analysis software (2021.2.1 version, Addinsoft, Paris, France) was used to perform the principal component analysis (PCA).^10^ Statistical evaluation of data was performed by considering only those analytes that were present in at least two samples.

## 3 Results and Discussion

The current study focused on the characterization of polyphenolic profiles of fifteen ancient varieties of apples cultivated within a small area of “Parco Nazionale del Cilento e Vallo di Diano” and the “Comunità Montana del Vallo di Diano”, flagship of the Campania Region. Five varieties of apples (*Milo Limungieddo Iaccio, Milo Limungieddo Gintilo, Milo Limungieddo Pizzuto, Limungieddo Santomichele iaccio,* and *Milo Limungieddo Rosa*) were probably derived from the *Limoncella*, a compact, juicy, and aromatic apple with white pulp characterized by a slightly acidic aftertaste Due to its exceptional organoleptic characteristics, it is considered among the most valuable southern apple cultivars even tasty for making an excellent cider. Other two varieties (*Milo Cinquiscocche Iaccio and Milo Limungieddo Iaccio*) derived from crosses between the *Limoncella* apple and *M. astracanica*, a species known in Italy since the 19th century. In Cilento also exists a variety called “Mela Iaccio” (iaccio = ice), not reported in this work, which has a translucent pulp when ripe and is derived from *M. astracanica*, described by Gallesio as follows: “an apple known in Tuscany under the name of *Mela Iacciola*, and that in Piedmont is mela dell’olio. I heard it call still mela diafana. Is this an apple which has the skin covered with large spots of a bright olive-green which penetrate the inside of the pulp, and give that part of the fruit an aspect and a very special sense”.^3^

All ancient varieties were subjected to a protocol of polyphenol extraction from both peel and pulp previous to the FC spectrophotometric assay and MS analyses (Figure 1). Two commercial apples, among the most cultivated varieties cultivated in Italy, Golden Delicious and Royal Gala were also analyzed to be compared with ancient varieties following the workflow shown in Figure 1.

### 3.1 TPC Analysis Using the FC Assay

**Figure 2:**
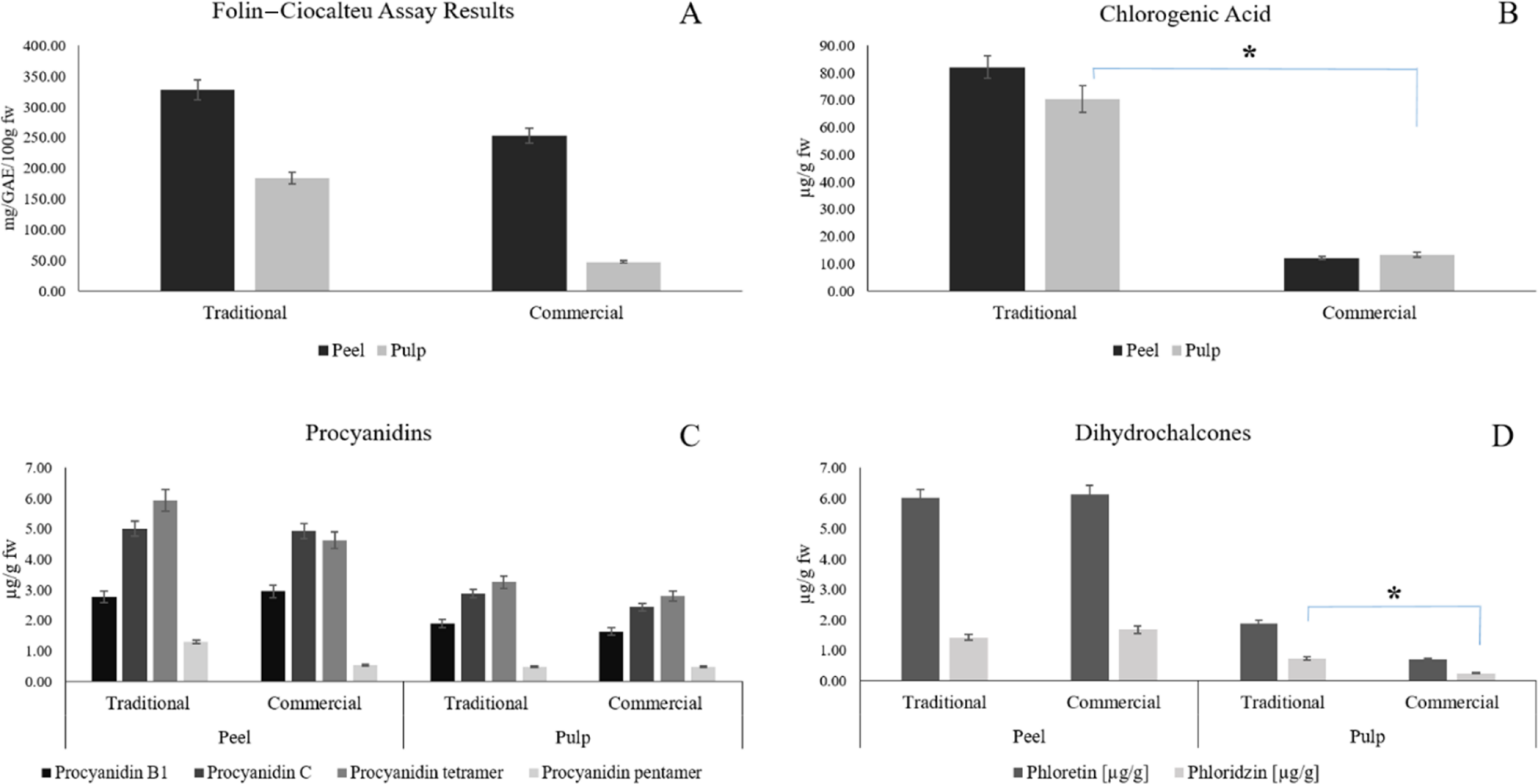
FC results for traditional and commercially available apple cultivars are reported in Panel A. The TPC values are expressed as mg GAE/100 g of FW for the peel and pulp. Chlorogenic acid average values are summarized in Panel B (μg/g FW). The average values of procyanidins (procyanidin B1, procyanidin C, procyanidin tetramer, and procyanidin pentamer), polymeric forms of catechins, are reported in Panel C (μg/g FW). The average concentrations of dihydrochalcones, the most abundant in apples, phloretin, and phloridzin are summarized in Panel D. Asterisk (*) denotes the samples with concentrations that are significantly different (*p* value ≤ 0.05) when comparing traditional and commercially available apples.

The FC assay is a commonly used test on vegetable matrices to determine the TPC.^11^ According to the Materials and Methods section, the methanol/water/formic acid (80/19/1) extracts from raw apple pulps and peels were added to the FC reagent and analyzed using a spectrophotometry assay, recording the absorbance at 765 nm. The results of the FC assays carried out on apple peels and pulps of both traditional and commercially available apple cultivars are summarized in Figure 2 (Panel A). TPC is expressed as milligrams of GAE/100 g of apple FW. The average values of the TPC for traditional apple varieties were 327.8 and 184.2 mg GAE/100 g FW for the peel and pulp, respectively, while 253.0 and 47.3 mg GAE/100 g FW were determined for the peel and pulp of commercial apples (Figure 2, Panel A). Apple peel was found to be richer in TPC than apple pulp in both traditional and commercial apple varieties, except for *Milo Limungieddo Puntiato* (Table S2 and Figure S1). The finding of a higher TPC in the peel has been similarly observed by others,^12^ and it is related to the well-known role of polyphenols in the defense of the fruit against pathogens and radiations.^13^ As also reported by Wolfe et al. (2003)^14^ apple peel is an important source of antioxidants and has higher amounts of phenolic compounds, antioxidant activity, and antiproliferative activity than the pulp.

Among the fifteen ancient apple cultivars, *Milo Cardarella* showed the highest TPC values in both the peel and pulp, while *Milo Cinquescocche Iaccio*, *Milo Limungieddo Iaccio*, *Milo Pizzente*, and *Milo Furticieddo* varieties, equivalent TPC values were recorded for the peel and pulp. Similar results to those reported in the present study were found in other published papers^15,16^ in which traditional and commercial apple varieties were compared and in which the extraction of polyphenols was carried out by using other extraction techniques (maceration or micro-matrix solid-phase dispersion). Lo Piccolo et al. analyzed the phenolic profiles and the total antioxidant activity of nine ancient apple cultivars in Garfagnana (Tuscany, Italy). These cultivars are locally produced and consumed, and their cultivation is very limited or abandoned. The authors provided the clear evidence that these ancient cultivars of apples should be revalued, both for local consumption and as a source of genetic variability for organoleptic and nutraceutical properties. These ancient cultivars had a higher total content of polyphenols in the pulp than commercial cultivars, as well as higher antioxidant activity.^9^

### 3.2 MRM/MS Analyses of Apple Peel and Pulp Extracts

Colorimetric assays such as the FC assay are found to be extremely effective, economical, and rapid in determining the TPC in food matrices. One of the main problems related to the FC assay is the possibility of interfering species that can undermine the correct determination of TPC. Among the main interfering species, there are sugars and free amino acids such as tyrosine and ascorbic acid, and these compounds interfere with the electron-donating capacity of the phenolics or, as in the case of tyrosine, absorb at the same wavelength, giving misleading enhanced absorption.^17^ A more accurate molecular characterization was performed by using a LC–MS/MS method in the MRM ion mode to detect and quantify each polyphenolic compound by monitoring specific transitions of each molecule. The great difference between this technology based on MS and the colorimetric assays is related to its targeted nature, which allows the identification and quantification of polyphenols not based on their total content but on specific fragmentation reactions for each molecule. MRM/MS analysis allowed a deeper knowledge of the polyphenolic raw extracts, empathizing the differences in the polyphenolic fraction in terms of molecules and amounts between traditional apple cultivars and modern ones. MRM/MS analyses of peel and pulp apple extracts were performed in triplicate, and the data analysis was carried out by using Skyline software. Results are summarized in the Supporting Information (Table S2), and as an example, Figure S1 reports the MRM chromatogram recorded for *Milo Verde* peel extract. During a single chromatographic run, 55 analytes form five main groups: phenolic acids, flavonols, anthocyanins, catechines and dihydrochalcones were monitored in each sample. Chlorogenic acid, the ester of caffeic acid with quinic acid, was determined as one of the most abundant compounds in apple peels and pulps, reaching concentrations on the scale of micrograms per gram of FW. This phenolic acid is one of the most ubiquitous dietary polyphenolic compounds, mainly found in coffee beans, tea, cocoa, berries fruits, citrus fruits, apples, and pears. As is widely known, chlorogenic acid shows numerous health benefits, such as anti-obesity, antidiabetic, anti-inflammatory, and antihypertension effects.^18^ In Figure 2, Panel B, the amount of chlorogenic acid in apple peels and pulps was reported as the average between traditional and commercial apple varieties. Significant changes (*p* value = 0.038) were observed by comparing traditional and modern apple pulps, as reported in Figure 2, Panel B. As can be inferred from Figure 2, Panel B, ancient cultivars are characterized by a higher content of chlorogenic acid in the peel than that in the pulp.^18^

More studies reported polyphenolic analysis in ancient Italian apple cultivars: Belviso et al. have analyzed the polyphenolic profiles of some ancient cultivar apples grown in Piedmont (Italy).^19^ Some of them have been found to be an interesting source of bioactive compounds when compared with the commercial variety Golden Delicious. In addition, they showed that some phenols such as chlorogenic acid and phloridzin, considered characteristic to the apple, are influenced by the year of collection. This finding is the first noteworthy difference to endow a higher nutraceutical value to ancient apples.

The analysis of catechin and its polymers showed that these compounds are mainly present in the peel of the fruit rather than in the pulp (Figure 2, Panel C). This result is compatible with the role played by procyanidins in fruits as they act as a defense against biotic and abiotic stressors. Particularly, their astringency protects fruits from pathogens and predators.^20^ Catechin was detected in every analyzed sample, and its average content for peels of ancient apples was 103.2 μg/g FW, which is similar to the values obtained for commercially available apples. A different finding was recorded for the catechin polymerization: a reaction whose extent is variable according to the part of the fruit (peel or pulp) or of the plant involved. In ancient apple cultivars, the procyanidin tetramer (procyanidin C) is the most abundant, while in Royal Gala and Golden Delicious, the trimer and tetramer are comparable. The only exception is *Milo Cardarella*, which was found to be the richest traditional apple cultivar for many of the target analytes (Table S2 and Figure S3).

Two dihydrochalcones typically found in apples, phloretin and its glycosylated form, phloridzin, were detected in almost all samples. Contrary to Giormaro et al.,^21^ from the histograms reported in Figure 2, Panel D, phloretin was found to be always more abundant than phloridzin in both traditional and commercially available apple cultivars, probably due to a partial hydrolysis of the glycoside form to the aglycone during the sample handling. No significant changes in phloretin concentrations were recorded in both apple peels and pulps, while phloridzin showed a *p* value of 0.05 when comparing ancient and modern apple pulp amount. Moreover, both compounds have a higher concentration in the apple peel than in the pulp. The exceptions to this finding are *Milo Limungieddo Iaccio, Milo Limungieddo Santomichele Iaccio*, and *Milo Limungieddo Puntiato*, where phloretin was not detected in the apple peel and where phloridzin levels in the pulp are higher than that in the peels (Table S2). The *Milo Limungieddo* family, which includes almost 30% of the analyzed samples, showed a lower amount of dihydrochalcones than the other apples in both the peel and pulp. Dihydrochalcones are currently employed for cosmetic and pharmaceutical purposes as they show high antioxidant, antidiabetic, immunomodulatory, antiviral, cardio, and hepatoprotective activity. These compounds can be either synthetized or extracted from plants.

Quercetin, another compound notoriously found in apples, was detected in all the analyzed samples. The concentration of quercetin and its derivatives in the peel is higher than that measured in the pulp. In Figure S4, the trend of quercetin and its derivatives is reported for apple peels. In ancient apples, levels of these molecules were lower than those in modern apple cultivars (an average value of 3.82 μg/g FW for the old varieties against 12.70 μg/g FW for the modern ones). Among the derived forms, quercetin rhamnoside is the most abundant. In contrast with the previous observations on glycosylated forms of polyphenols, a higher concentration of quercetin-3-*O*-rhamnoside was observed in modern apples than that in ancient ones, always respecting the trend of peels being richer than pulps (Figure S4).

The LC–MS/MS method used for polyphenol characterization contained 13 different anthocyanins. Among them, petunidin-3-*O*-glucoside and peonidin-3-*O*-glucoside (Figure S5) were detected and quantified in all apple samples.^16^ Although these molecules are not considered primary pigments in apples, their abundance could be explained by the color of the treated samples (Figure 1). Since the 1960s, it has been known that the red pigmentation of apple peels is associated with the presence of cyanidin and its derivatives,^22^ but none of the analyzed apples were characterized by dark shades of red that are usually associated with these pigments. Peonidins cause orange-red coloring, which is compatible with the pigmentation observed for the analyzed apple cultivars.^23^

These anthocyanins are more abundant in peels than in the pulp. By comparing traditional and commercial apple varieties, with the exceptions of *Milo Rose* and *Milo Pizzente*, petunidin-3-*O*-glucoside and peonidin-3-*O*-glucoside presented a concentration two to five times higher than that in Royal Gala and Golden Delicious (Figure S5).

The overall results obtained by targeted analysis showed that most of the analyzed polyphenols are more abundant in apple peels rather than in the pulp, confirming what was already well-known about the defensive role of fruit peels and their high content of nutrients. Moreover, ancient cultivars proved to be incredibly rich in anthocyanins, dihydrochalcones, and chlorogenic acid, whose benefits and antioxidant effects are widely known. Even D’Abrosca et al. demonstrated higher levels of total phenols and total flavonoids in the apple peel, verifying previous studies that highlighted their protective role against ultraviolet radiation and as a chemical defense against pathogens and predators of this molecule class.^24^

### 3.3 Principal Component Analysis

**Figure 3:**
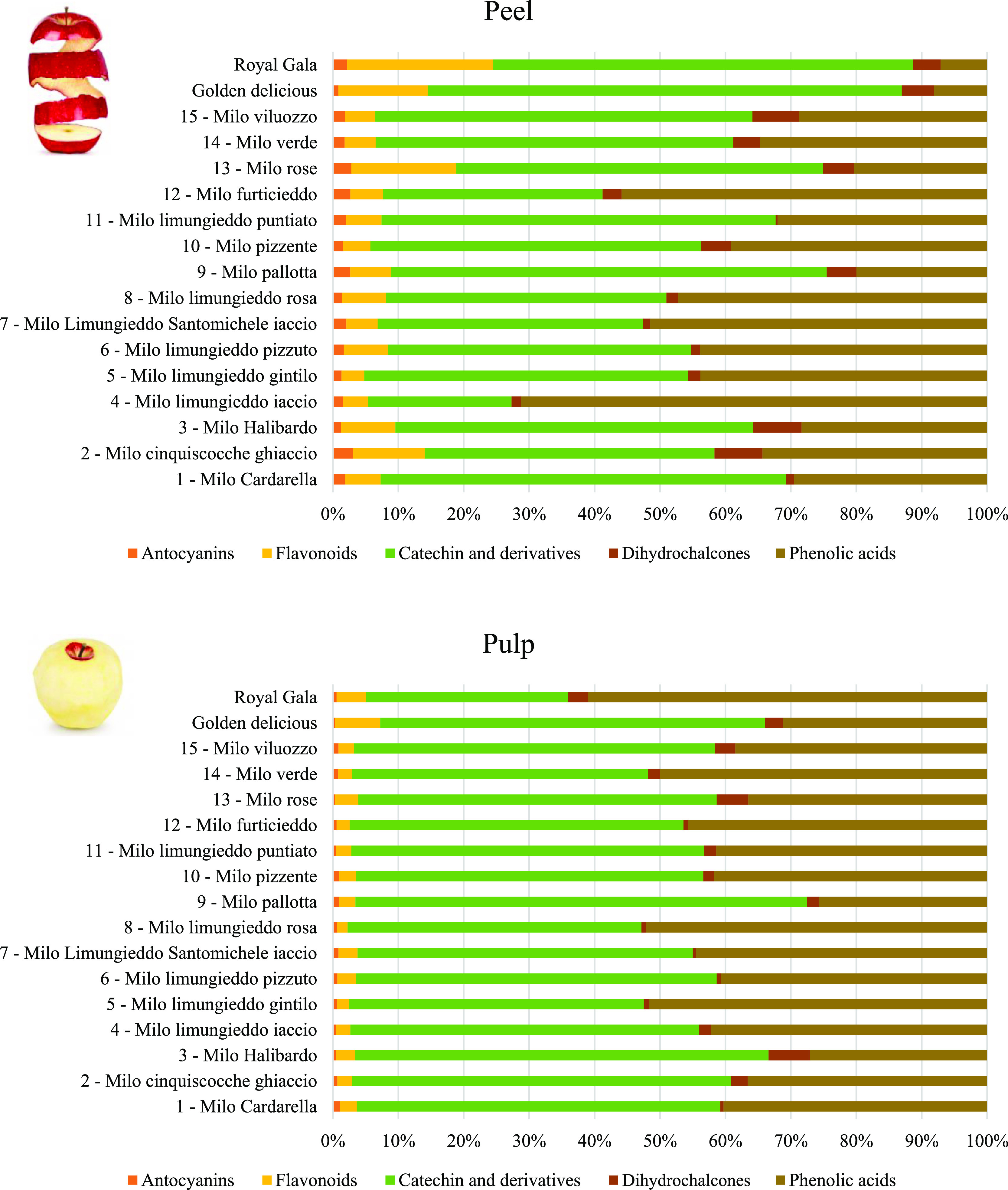
Peel and pulp compositions of modern and ancient apples, reporting the percentage of each class: anthocyanins, flavonoids, catechin and derivatives, phenolic acids, and dihydrochalcones.

The LC–MS/MS analysis of the peel and pulp extracts allowed us to outline five main classes of polyphenols characterizing both modern and ancient apples. The peel and pulp compositions of modern and ancient apples, summarizing the percentage of each class including anthocyanins, flavonoids, phenolic acids, catechin and derivatives, and dihydrochalcones, are described in Figure 3.

**Figure 4:**
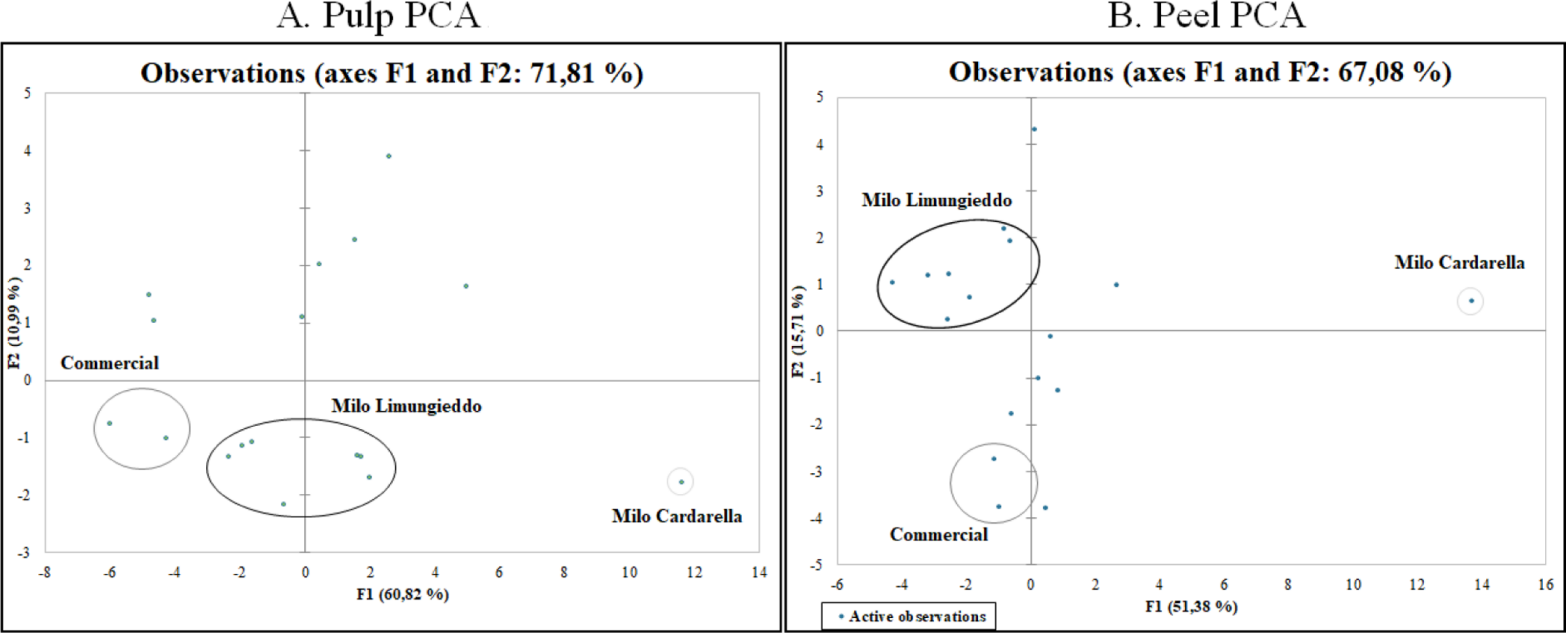
Two-dimensional PCA for the pulp (Panel A) and peel (Panel B), showing associations between experimental samples and all monitored and quantified polyphenol compound abundance. The diagrams display the first and second principal factors (PC 1 and PC2, respectively) of the PCA.

PCA, a multivariate statistics-based detection method, was used to reduce the dimension of or transform multiple indicators into a few comprehensive ones for extracting features and revealing the relationship between variables.^25^ To highlight differences between old and new apple varieties according to their peel and pulp polyphenol content, PCA was performed by using data obtained from MRM/MS quantification (Figure 4).

The first two principal components make up 60.8 and 10.9% of the total variance contribution ratio for the apple pulp and 51.4 and 15.7% for the apple peel. Ancient and new apple varieties occupy relatively independent spaces in the distribution map, suggesting a differentiation between different apple cultivars.

Polyphenol profiles for the apple pulp and peel were quite similar, but interesting information was extracted from the PCA:

- *Milo Cardarella* is an outlier, as previously demonstrated by comparing the amount of some molecules across the varieties.
- *Milo Limungieddo* varieties are grouped in the same region of the distribution map in pulp and peel PCA.
- Pulp and peel PCA for Royal Gala and Golden Delicious varieties showed the clusterization of these two varieties in the same region of the map as well as *Milo Rose, Milo Halibardo*, *and Milo Verde.*

Positive characteristics were found in the ancient apple varieties in comparison with commercially available ones. Most apple varieties contain more polyphenols in the peel than in the pulp, and ancient cultivars showed higher TPC and polyphenol content. As a whole, this study highlighted the immense richness of these products in terms of content of substances with a proven beneficial action on health. It can also be considered a good starting point for the re-evaluation of these varieties for a larger-scale cultivation and in the development of new functional foods.

## 4 Conclusions

The goal of the present paper was focused on the application of an MS-based method (MRM/MS) to identify and quantify polyphenols in traditional and modern apple varieties.

The obtained results derived from the comparison of MS data between two commercial apples and some ancient apple varieties from Cilento (Campania) pointed out some considerations:

- the traditional apple peels had 1.65-fold and 6.5-fold higher anthocyanin and phenolic acid contents compared to commercially available peels, respectively;
- the content of all the classes of investigated molecules was at least two-fold higher than that of Royal Gala and Golden Delicious pulps.

These findings highlighted the greater concentration of polyphenols in ancient varieties than that in the commercially available ones both in the peels and in the pulps, paving the way for the revaluation of varieties that are widespread only in restricted geographical areas.

In conclusion, the richness of these ancient cultivars in terms of genetic variability and content of polyphenols of nutraceutical relevance should encourage their diffusion, revaluation, and cultivation not only for local consumption.

## Supporting Information Available

MRM/MS method in positive ion mode; TPC of ancient apple cultivars determined using the FC assay and expressed as mg GAE/100 g of FW; MRM chromatograms of the monitored transitions of *Milo Verde* peel raw extract; procyanidin levels (μg/g) in peels and pulps for traditional and commercial apple varieties; quercetin and quercetin derivatives levels (μg/g) quantified in apple peels using LC–MS/MS; and petunidin-3-*O*-glucoside and peonidin-3-*O*-glucoside levels (μg/g) in apple peels and pulps (PDF)
Quantitative analysis of target polyphenols in ancient and commercially available apple cultivars (XLSX)
