# Supplementary material for: LC–MS/MS-Based Quantification Method of Polyphenols for Valorization of Ancient Apple Cultivars from Cilento
Source: ACS Food Sci Technol. 2022 Mar 30;2(4):647–54. doi: 10.1021/acsfoodscitech.1c00439 (PMC9016709; doi:10.1021/acsfoodscitech.1c00439)
Supplement: Supplementary file 1 — fs1c00439_si_001.pdf [file fs1c00439_si_001.pdf]

LC-MS/MS-based quantification method of polyphenols for valorisation of ancient apple cultivars from Cilento

Anna Illiano[1,2,3], Gabriella Pinto[1,3], Maria Antonietta Carrera[1], Angelo Palmese[6], Riccardo Di Novella[4], Paolo Casoria[5], Angela Amoresano[1,3]

1. Department of Chemical Sciences, University of Naples Federico II, 80126 Naples, Italy.

2. CEINGE Advanced Biotechnologies, University of Naples Federico II, 80145 Naples, Italy.

3. INBB, Istituto Nazionale Biostrutture e Biosistemi, Consorzio Interuniversitario, 00136 Rome, Italy.

4. Ecomuseo della Valle delle Orchidee e delle Antiche Coltivazioni-Sassano (Sa)-PNCVDA.

5. Department of Sciences and Technology, University of Naples Parthenope, 80143 Naples, Italy.

6. Pharmaceutical & Analytical Development Biotech Products, Merck Serono SpA, an affiliate of Merck KgaA, Darmstadt, Germany , Roma , Italy

Corresponding author: gabriella.pinto@unina.it

Keywords: Ancient apple cultivars; Mass spectrometry; Polyphenols; Campania region

Table S1: MRM/MS method in positive ion mode.

Molecule	Precursor ion (m/z)	Product ion (m/z)	DP (V)	EP (eV)	CE (eV)	CXP (eV)
Delphinidin diglucoside	627	303	90	14	46	32
		256				
Cyanidin-3,5-di-O-glucoside	611	287	177	14	55	19
Delphinidin-3-O-glucoside	465	303	181	14	30	21
		285				
Cyanidin-3-O-glucoside	449	287	192	12	27	20
		241				
Delphinidin-3-O-arabinoside	435	303	192	10	25	21
		285				
Petunidin-3-O-glucoside	479	317	172	13	27	21
		274				
Cyanidin-3-O-arabinoside	419	287	194	11	24	19
		241				
Pelargonidin-3-O-glucoside	433	271	163	10	28	24
Peonidin-3-O-glucoside	463	301	104	11	29	20
		268				
Malvidin-3-O-glucoside	493	331	94	12	29	21
		270				
Malvidin-3-O-arabinoside	463	331	94	11	24	22
		270				
Delphinidin rutinoside	611	303	165	14	45	21
		284				
Malvidin 3-O-p-coumaroylglucoside	639	331	121	6	29	24
		315				

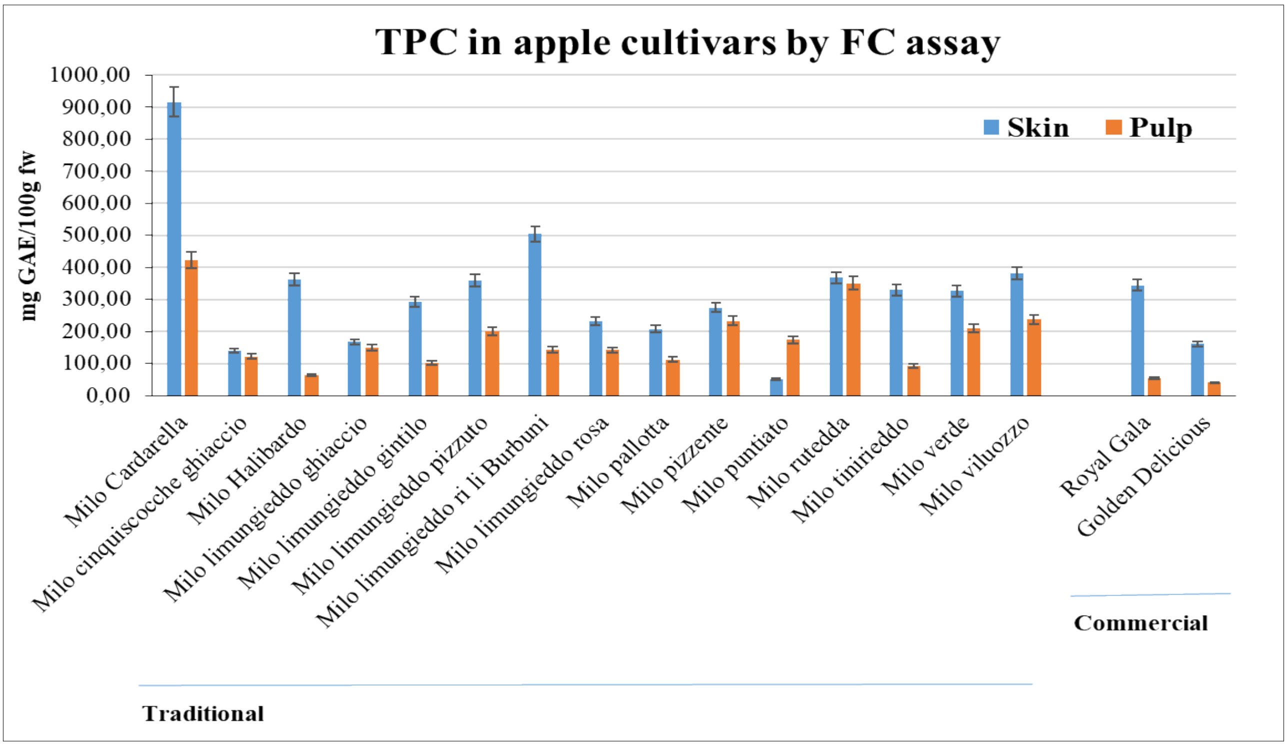

SF1. TPC of ancient apple cultivars determined by FC assay and expressed as mg GAE/100g of fresh fruit (FW)

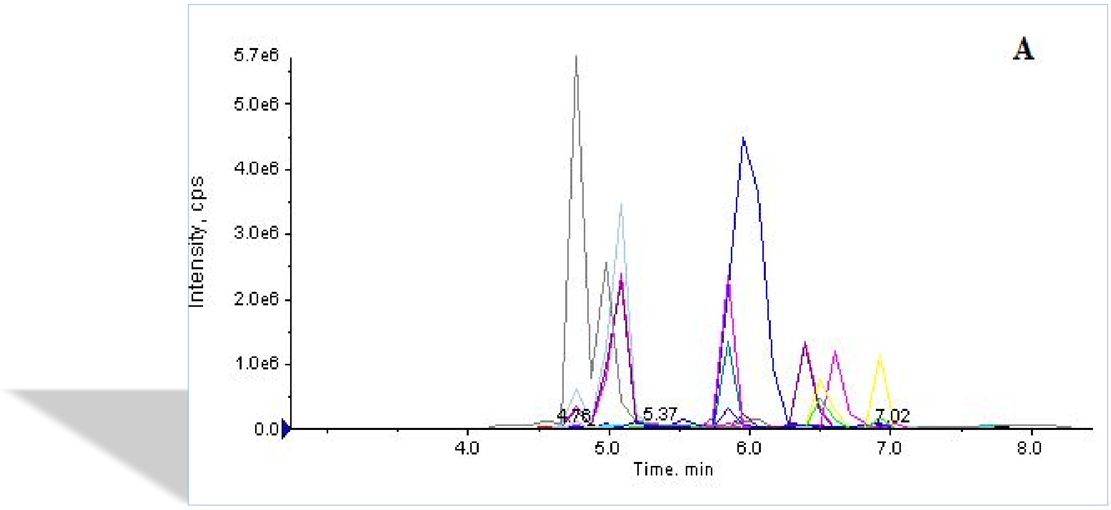

SF2. SF2. MRM chromatogram of the monitored transitions of *Milo Verde* peel raw extract is reported.

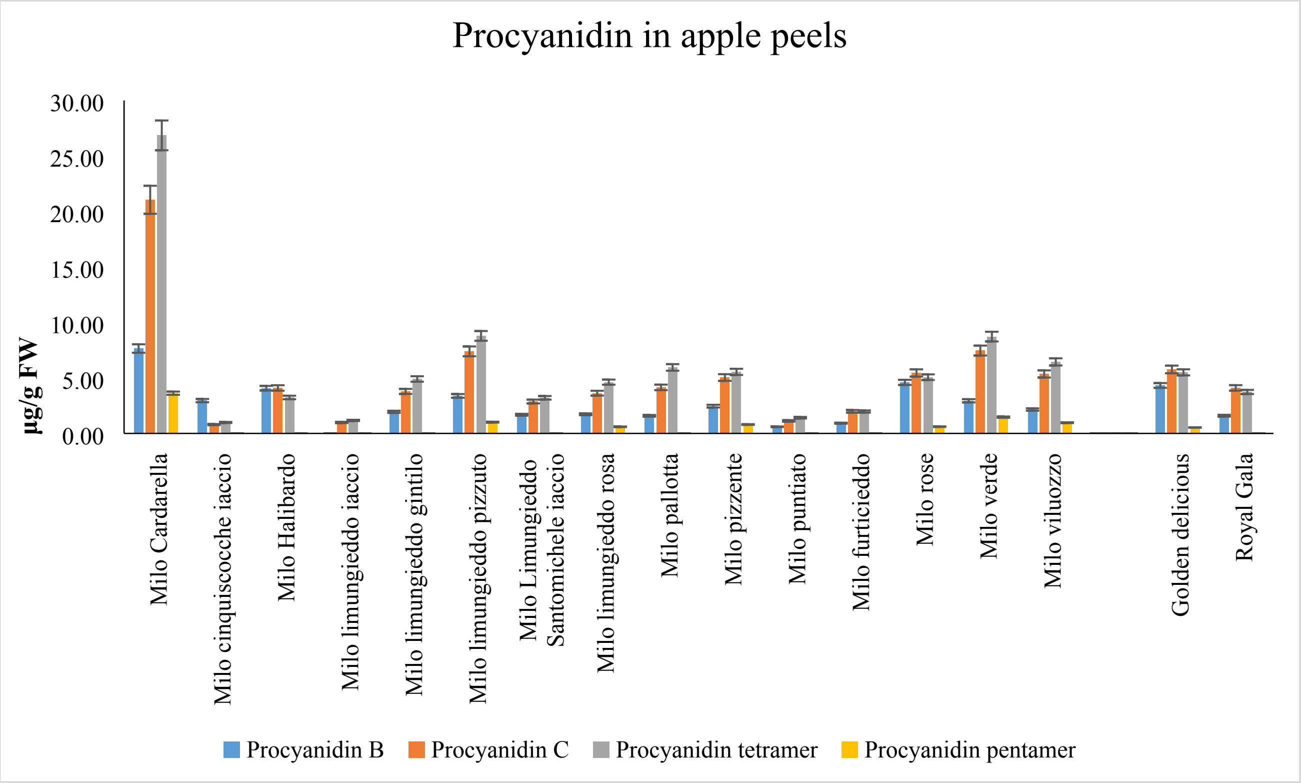

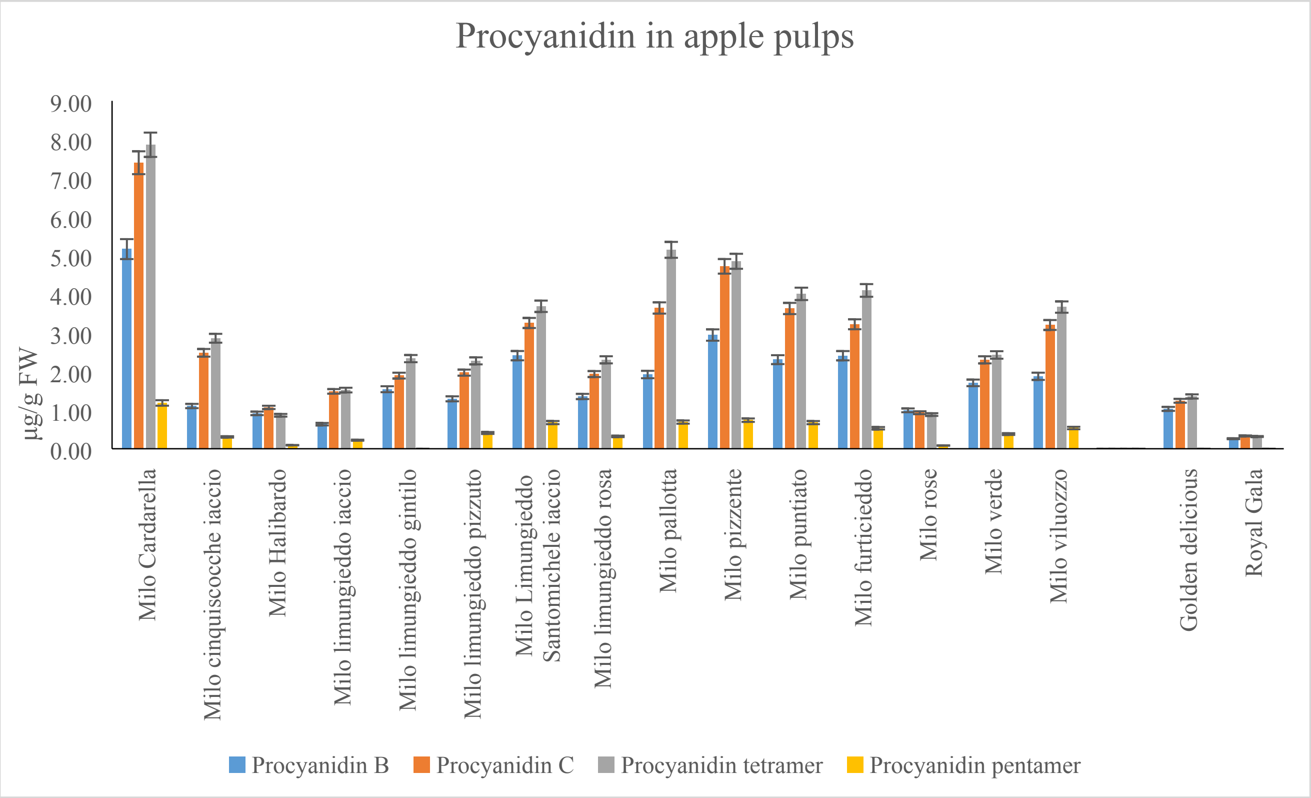

SF3. Procyanidin levels (µg/g FW) peels and pulps for traditional and commercial apple varieties.

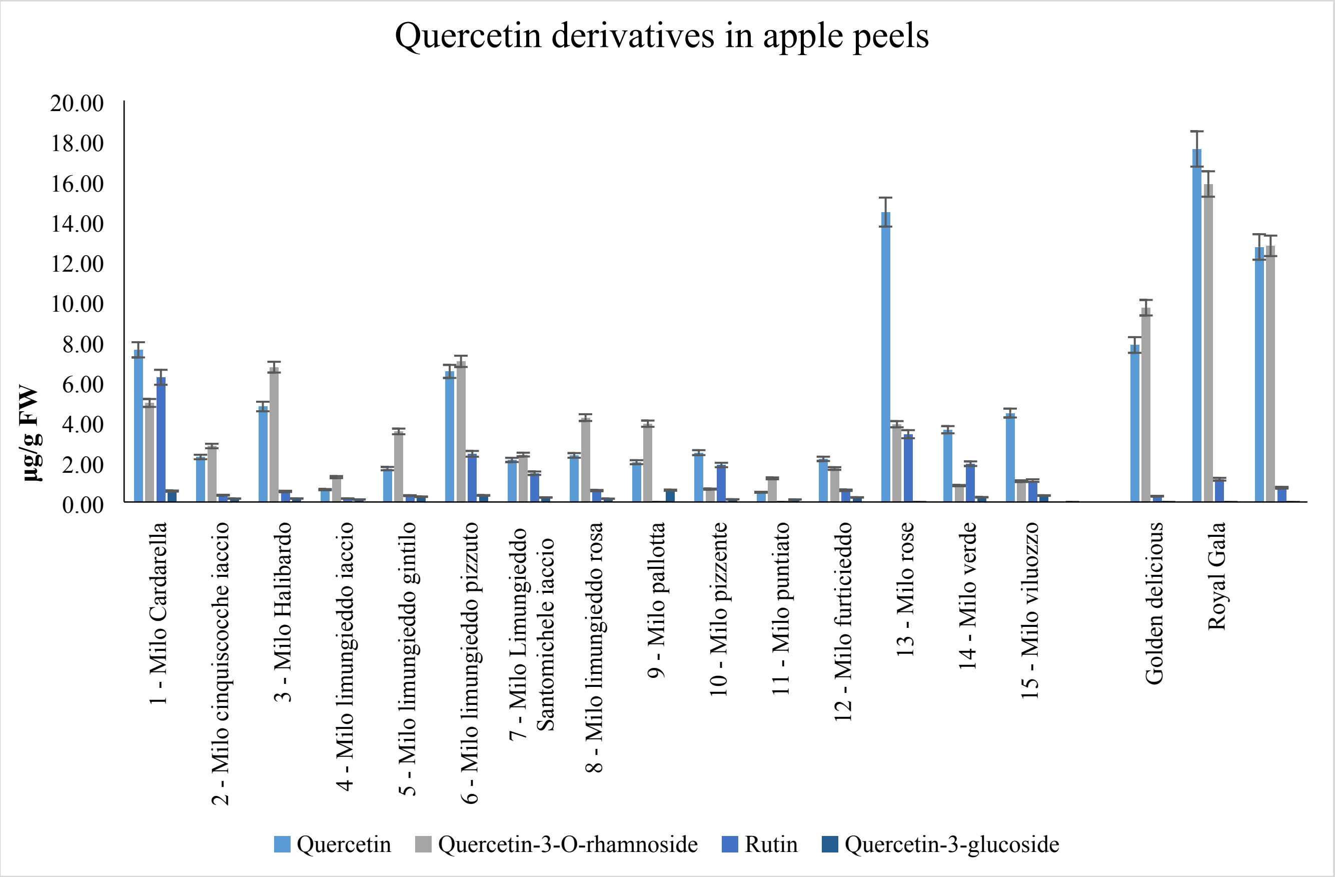

SF4. Quercetin and quercetin derivatives levels (µg/g) of fresh fruit (FW) quantified in apple peels by LC-MS/MS.

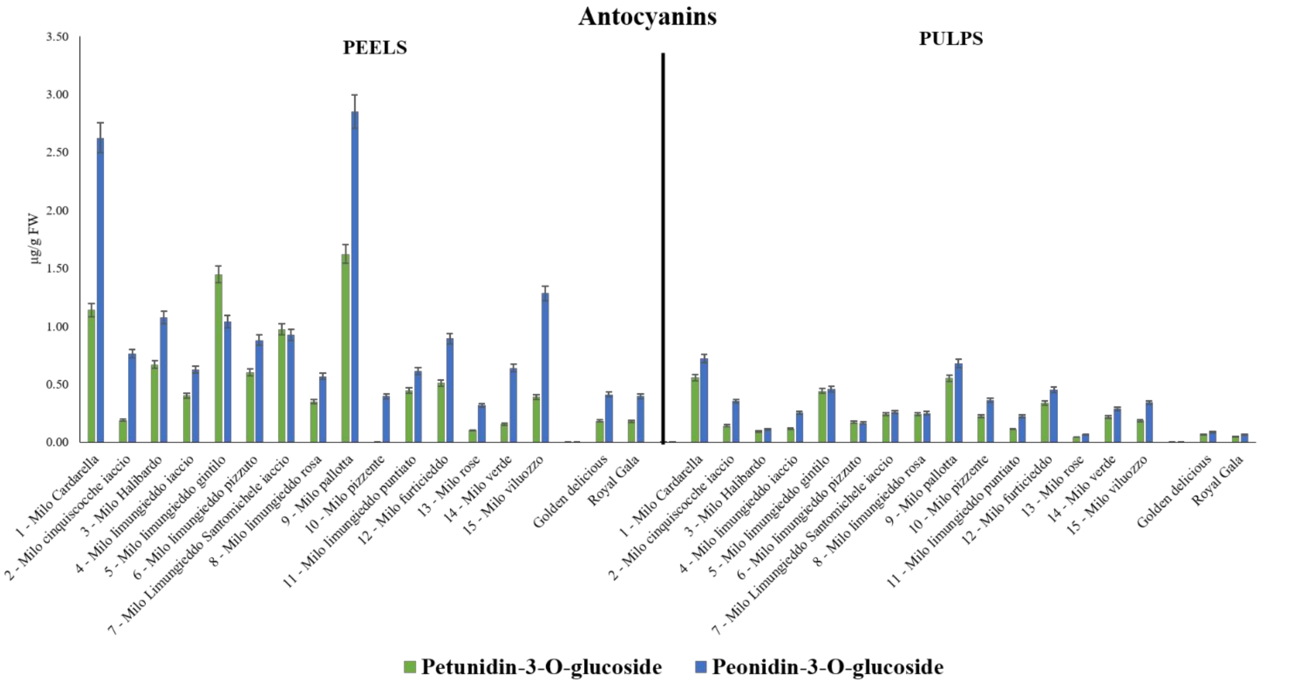

SF5. Petunidin-3-O-glucoside and peonidin-3-O-glucoside levels (µg/g FW) in apple peel and pulps.
